# Livestock grazing intensity affects abundance of Common shrews (*Sorex araneus*) in two meadows in Denmark

**DOI:** 10.1186/1472-6785-9-2

**Published:** 2009-01-20

**Authors:** Niels M Schmidt, Henrik Olsen, Herwig Leirs

**Affiliations:** 1 Section for Climate Effects and System Modelling, Department of Arctic Environment, National Environmental Research Institute, University of Aarhus, Frederiksborgvej 399, PO Box 358, DK-4000 Roskilde, Denmark; 2 Section of Zoology, Department of Ecology, Faculty of Life Sciences, University of Copenhagen, Thorvaldsensvej 40, DK-1871 Frederiksberg C, Denmark; 3 Danish Pest Infestation Laboratory, University of Aarhus, Faculty of Agricultural Sciences, Department of Integrated Pest Management, Skovbrynet 14, DK-2800 Kongens Lyngby, Denmark; 4 Evolutionary Ecology Group, Department of Biology, University of Antwerp, Groenenborgerlaan 171, B-2020 Antwerpen, Belgium

## Abstract

**Background:**

Current nature conservation in semi-natural grasslands often includes grazing and hay cutting, as well as the abandonment of draining. Semi-natural grassland and in particular meadows constitute important habitat type for a large number of animal species in today's fragmented and intensively cultivated landscape of Europe. Here we focus on the population characteristics of Common shrews *Sorex araneus *in relation to livestock grazing intensity in two wet meadows in western Denmark.

**Results:**

High grazing intensity had a significant negative effect on Common shrew number compared to low grazing intensity and no grazing. Common shrew abundance was generally, but not significantly, higher on the low grazing intensity plots than on the ungrazed controls. No differences in body mass, sex ratio, or reproductive output between Common shrew individuals from the various grazing treatments were found.

**Conclusion:**

No negative effects of low intensity grazing on Common shrew abundance were found compared to the ungrazed control. Low intensity grazing thus seems a suitable management regime for Common shrews, when grazing is needed as part of the meadow management scheme. High intensity grazing on the other hand is not a suitable management tool.

## Background

In Denmark as well as in most other European countries, the amount of land covered by semi-natural grassland has decreased dramatically during the 20^th ^century concurrent with the general intensification of the agricultural production. To reverse this trend, actions are being taken in many places to either maintain or re-establish this biotope, and in particular, the meadow community. Today's nature conservation is a return to the old extensive agricultural methods, and includes grazing and hay cutting, as well as the abandonment of draining. Semi-natural grassland and in particular meadows constitute important habitat types for a large number of animal species in today's fragmented and intensively cultivated landscape in Europe.

Hay cutting and livestock grazing is known to affect a number of organisms, but the response to grazing may vary across classes of organisms and with the intensity of grazing [[1], and references therein]. The effect of haying and grazing on plant diversity and composition is well-documented [e.g. [2-4]]. Also, many avian species may respond to grazing, and certain grazing intensities may favour some species over others [e.g. [5]]. In contrast to this, only limited data on the response of the mammalian vertebrates to the application of these traditional farming methods is available [but see [6]]. However, meadow management in general may reduce small mammal species richness [7,8]. Apart from being simple disturbances induced onto the flora and fauna, grazing and haying may change the physical environment, the plant composition and height. This may in turn influence the spatio-temporal distribution of the small mammals, and small mammal biomass has been found to decreases with grazing intensity [8,9].

The Common shrew (*Sorex araneus*) exploits a variety of terrestrial as well as semi-terrestrial habitats [10]. Though today's meadow management does not aim specifically at improving Common shrew habitats, the species is one of the most common mammals on meadow communities, and may play an important role in the trophic interactions in this biotope. In the present study, we therefore focus on the Common shrew in order to reveal the impact of three different grazing regimes applied in meadow management on the population characteristics of this species.

## Results

During the entire trapping period we caught 570 individual Common shrews. Pygmy shrews *S. minutus *and Water shrews *Neomys fodiens *were also caught, but only in small numbers.

**Figure 1:**
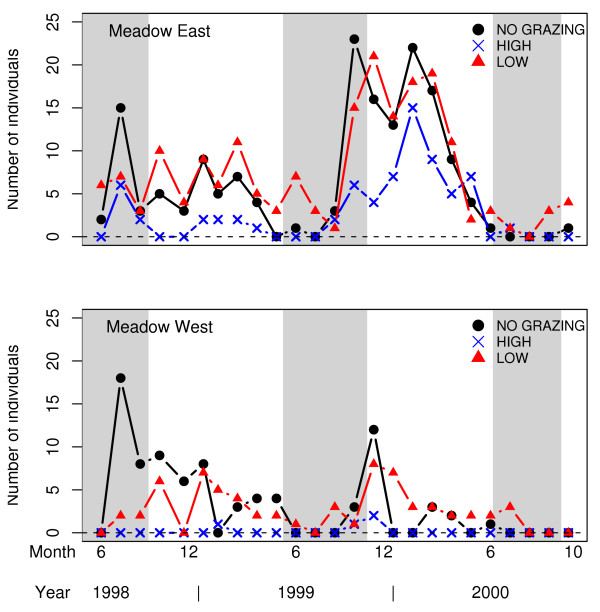
**Common shrew numbers in relation to livestock grazing intensity**. The number of Common shrews *Sorex araneus *trapped on meadow East and meadow West during the study period. Grey bars indicate periods with livestock grazing.

In all six trapping plots, the number of individual Common shrews caught in each trapping session showed large fluctuations among trapping sessions as well as inter-annually (Figure 1). The autoregressive component was not significant in the model (P > 0.05), whereas trapping session nested within year was significant (P < 0.05). The number of Common shrew individuals varied significantly among meadows (F_1,4.65 _= 28.62, P = 0.0038) and among grazing treatments (F_2,4.65 _= 18.76, P = 0.0059). Meadow East held more Common shrews than meadow West, and the high grazing intensity treatment (HIGH) held significantly fewer Common shrews than the ungrazed control (NO GRAZING) (P = 0.018) and the low intensity grazing treatment (LOW) (P = 0.006). Common shrew numbers in LOW and NO GRAZING did not differ significantly (P = 0.401).

The body mass corrected for uterus and testes mass varied seasonally, but body mass did not vary significantly with either trapping session or year, and these two were therefore excluded in the following analyses. Grazing treatment did not affect Common shrew body mass significantly (F_2,438 _= 2.05, P = 0.1303; Table 1), but Common shrew body mass was significantly higher on meadow East than on meadow West (F_1,440 _= 2.45, P = 0.0147; Table 1).

**Table 1:** Common shrew population characteristics on two Danish meadows with three livestock grazing intensities.

Meadow	Treatment	Number trapped	Number autopsied	Mean number of placental scars		Mean number of fetuses		Mean female body mass (g)		Mean male body mass (g)	
East	NO GRAZING	163	156	4.67	[3.06]	7.67	[0.82]	7.60	[1.24]	7.45	[1.30]
	LOW	186	144	-	-	5.50	[2.38]	7.23	[1.23]	7.16	[1.15]
	HIGH	71	56	6.00	-	6.33	[1.54]	7.54	[1.41]	7.20	[0.99]
West	NO GRAZING	81	49	-	-	8.00	-	6.99	[1.13]	7.03	[0.86]
	LOW	65	60	7.50	[0.71]	6.50	[0.71]	7.46	[2.28]	6.99	[1.24]
	HIGH	4	0	-	-	-	-	-	-	-	-

Data on placental scars, foetuses and body mass are from autopsied adult individuals only. Numbers in brackets are standard deviations.

During the entire trapping period, the overall sex ratio was close to unity (52% males, 48% females; χ^2 ^= 0.2523, P = 0.6154). No correlation between female corrected body mass was found on any of the treatments (F_1,9 _= 2.66, P = 0.1543), and no statistically significant differences in the number of foetuses or uterine scars among grazing treatments was found (F_2,6 _= 1.12, P = 0.3319; Table 1).

## Discussion

The three grazing treatments applied on the two meadows affected the population dynamics of the Common shrews markedly, and despite the overall differences in Common shrew numbers between the two meadows, high intensity grazing always resulted in significantly lower Common shrew numbers compared to both low intensity grazing and no grazing. Areas with low grazing intensity often held more Common shrews than the ungrazed control, though the overall differences in abundance were not statistically significant (Table 1). The pattern in the Common shrew trappings is very similar to that observed for field voles *Microtus agrestis *on the very same meadows [11]. Thus, in the low intensity grazing treatment, the potentially negative impacts of grazing livestock, such as mechanical disturbance, were apparently fully compensated for by the positive effects of livestock grazing.

The similarity in the response to grazing intensity amongst two ecologically distinct species, a rodent and an insectivore, points to a common environmental parameter as driver of the population dynamics. Schmidt et al. [11] suggested that for field voles the observed pattern was primarily due to livestock grazing creating a more heterogeneous vegetation in the low intensity grazed areas as compared to areas with high grazing intensity or no grazing, which, in case of the Common shrew, fully compensate for the potentially negative effects of grazing, such as mechanical disturbance. As for the field voles [11], vegetation cover, and, thus, risk of predation, is a probable cause of the grazing treatment effects observed on Common shrews in the two meadows [see also [8]]. Additionally, grazing may affect plant species composition, and livestock trampling may create a more heterogeneous micro-topographic environment, which, in turn, may affect the composition and availability of invertebrates. Increasing grazing intensity is generally believed to be accompanied by decreasing invertebrate abundance and species numbers [e.g. [12]]. Roberts & Morton [13], however, reported that Scarabaeidae biomass peaked an intermediate grazing intensity. Also, invertebrate species richness may benefit from low intensity grazing [14]. Shrews generally adapt rapidly to spatial and temporal changes in prey availability [10], and the observed pattern of Common shrew abundance found in this study may, thus, be attributed to indirect effects of livestock grazing affecting the distribution of Common shrew food.

Unlike for the field voles [11], we found no qualitative differences between Common shrew individuals caught in the three grazing treatments. That is, no differences in body mass, reproductive output, or sex ratio between treatments. The only qualitative difference we found was between individuals from the two meadows studied, and individuals from the western meadow were lighter than individuals from the eastern meadow. This difference in body mass points out meadow West as being sub-optimal compared to meadow East. Generally, only few shrews were caught on this meadow, and generally the populations on meadow West fluctuated more irregularly compared to meadow East. Meadow West was generally more water-logged and flooded more often than meadow East, and may therefore be a less suitable and more unpredictable habitat than meadow East. Shrews are rapid colonisers [15], and Common shrew numbers on meadow West was therefore probably more determined by an unstable alternation between immigration and emigration, whereas the shrews on meadow East belonged to more stable populations. Recapture rates were, however, too low the verify this.

The consistent response of the insectivorous Common shrew and the rodent Field vole [11] to grazing intensity across these two, highly different meadows stresses low intensity livestock grazing as a highly suitable means in today's meadow management, at least in the short run. Long-term changes in meadow vegetation composition etc. induced by grazing livestock may either alter or consolidate the response of both rodents and insectivores to livestock grazing reported here.

## Conclusion

Livestock grazing intensity had marked effect on Common shrew numbers, and the highest number of Common shrews was found in the low intensity grazed treatments and the ungrazed controls, while the high intensity grazing treatments held the lowest number of Common shrews. Thus, when grazing is needed as part of the meadow management scheme, low intensity grazing seems suitable for Common shrews and small mammals in general [see [11]].

## Methods

### Study sites

The study sites were situated in two meadows in western Denmark (56° 29'N, 9° 49'E), approximately 4 km from each other and separated by a forested hill, fields and roads. Draining has been abandoned there since the 1980's, and at the time of the study, in 1998–2000, the meadows appeared as water logged. Several old canals still traversed the areas. Meadows were reseeded in 1988 and 1990, respectively. The vegetation on the meadow West was dominated by *Festuca rubra*, *Phleum pratense*, *Poa trivialis*, and Bryophytes. Meadow East was dominated by *Poa pratensis*. *Ranunculus repens *was the dominating herb on both meadows [4].

In 1997, several different grazing regimes were established on the meadows as part of a large multi-disciplinary study (Land use – The farmer as manager of the landscape). From summer 1998 to spring 2000 we conducted small mammal trappings on these different meadow management regimes [see [11]]. On each meadow, we had one pen with cattle grazing (4.8 steers per ha; maximum biomass = 1254 ± 300 kg per ha (mean ± SD); referred to as HIGH), and one pen with sheep grazing (4.5 ewes plus lambs per ha; 396 ± 10 kg per ha; referred to as LOW). Although we in a previous study showed that the effect of livestock grazing on the biomass of another small mammal, the Field vole, on these particular meadows was related to grazer biomass rather than livestock species [11], we recognize that the use of two livestock species may to some extent confound the treatment effect. The use of sheep and cattle in LOW and HIGH, respectively, may therefore, due to the different body mass and foraging behaviour of the two livestock species [see e.g. [6,16]], result in a larger difference between treatments than could be expected from livestock biomass alone.

Pens were grazed from mid May to mid October. Each pen covered approximately 1–2 ha. Due to the multi-disciplinary set-up, each pen was divided into two halves, and hay cutting was conducted on each half every two years in June – July, and grazed thereafter. The other half was grazed the entire period. The succeeding year the grazing and hay cutting regime on the two halves was reversed. Finally, we had one pen with hay cutting only (referred to as NO GRAZING). Again hay cutting on each half alternated between years.

### Trapping regime

**Figure 2:**
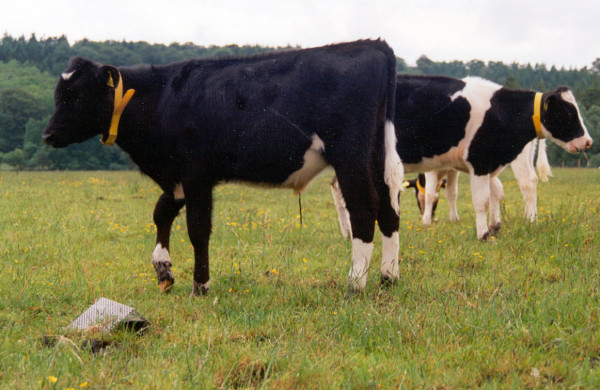
**Small mammal trap protected from livestock trampling**. A custom made perforated steel plate protected small mammal traps from livestock trampling, here on a pen with high grazing intensity.

On each treatment we placed a 6 × 6 Ugglan live trap square grid placed 10 m apart. Ugglan Lemming and Special traps alternated between lines. Traps were left unset on the grids between trapping sessions, and protected from mechanical disturbance of particularly cattle by a custom made perforated steel plate mounted over each trap (Figure 2). Traps were bedded with hay, and baited with rolled oats, apple and in some trapping sessions minced meat. We trapped for three days and nights (648 trap nights) every four weeks, and a total of 25 trapping sessions were conducted (16.200 trap nights). Traps were checked every 24 hours. The Common shrews examined in the present study were all caught in connection with a larger investigation targeted at examining mainly rodent population ecology and meadow management [11], and were a non-targeted species captured during that study. Common shrews that died in the trap were taken back to the lab and autopsied to obtain the body mass corrected for the mass of the uterus or testes, and to determine the number of uterine foetus or uterine scars. Live individuals were handled in the field, PIT-tagged (Francis Scientific Instruments, Cambridge, UK) for identification, and released immediately thereafter at the point of capture. All animal trapping and handling complied with Danish legislation and happened under permits of the Danish Pest Infestation Laboratory.

### Data analyses

We analysed the number of Common shrews in the various grazing treatments using an autoregressive General Linear Mixed Model (GLIMM) with the Log-transformed number of Common shrews as response variable and grazing treatment and meadow as fixed factor, while both trapping session and year were regarded as random. Trapping sessions were nested within year. As post hoc test we used Tukey-Cramer (P < 0.05). Variation in Log-transformed body mass of autopsied individuals (corrected for testes or uterus mass) were analysed using a similar mixed model approach. Model reduction was conducted using likelihood-ratio tests [17]. Variation in sex ratio was analysed by means of Fishers exact test.

## Authors' contributions

NMS participated in data collection, carried out the statistical analyses, and drafted the manuscript. HO participated in the data collection and contributed to the writing. HL designed the study and contributed to the writing. All authors read and approved the final manuscript.
